# Modern analogs for ammonia flux from terrestrial hydrothermal features to the Archean atmosphere

**DOI:** 10.1038/s41598-024-51537-2

**Published:** 2024-01-17

**Authors:** J. David Felix

**Affiliations:** Physical and Environmental Sciences Department, Center for Water Supply Studies, Texas A&M University – Corpus Christi, Corpus Christi, USA

**Keywords:** Astronomy and planetary science, Origin of life, Biogeochemistry, Element cycles, Planetary science, Atmospheric chemistry, Geochemistry

## Abstract

The isotopic composition of nitrogen in the rock record provides valuable evidence of reactive nitrogen sources and processing on early Earth, but the wide range of δ^15^N values (− 10.2 to + 50.4‰) leads to ambiguity in defining the early Precambrian nitrogen cycle. The high δ^15^N values have been explained by large fractionation associated with the onset of nitrification and/or fractionation produced by ammonia-ammonium equilibrium and water–air flux in alkaline paleolakes. Previous flux sensitivity studies in modern water bodies report alkaline pH is not a prerequisite and temperature can be the dominate parameter driving water–air flux. Here, I use the chemical and physical components of 1022 modern hydrothermal features to provide evidence that water–air NH_3_ flux produced a significant source of fixed nitrogen to early Earth’s atmosphere and biosphere. With regard to the modeled average NH_3_ flux (2.1 kg N m^−2^ year^−1^) and outlier removed average flux (1.2 kg N m^−2^ year^−1^), the Archean Earth’s surface would need to be 0.0092, and 0.017% terrestrial hydrothermal features, respectively, for the flux to match the annual amount of N produced by biogenic fixation on modern Earth. Water–air NH_3_ flux from terrestrial hydrothermal features may have played a significant role in supplying bioavailable nitrogen to early life.

## Introduction

Reactive nitrogen’s (N_R_) vital role in the origin of life and predicting the potential for life on other planets has led to decades of work investigating clues to N_R_’s origins and source magnitudes on early Earth. In contrast to modern environments where a primary N_R_ species is nitrate, early Earth’s reducing environments were dominated by ammonia/ammonium (NH_3_/NH_4_^+^), until the Great Oxidation Event (~ 2.3 Ga)^1^. Researchers have postulated several abiotic NH_3_ production scenarios including direct creation of NH_3_/NH_4_^+^ via hydrothermal and photochemical reduction or by fixation processes (e.g., volcanic, lightning) creating oxidized N species followed by reduction^2–5^. However, investigations into the potential magnitude of abiotic production mechanisms suggest they were too minor to sustain the Archean biosphere thus implicating an early biotic source^6^. Geological evidence and the presence of NH_4_^+^ enriched phyllosilicates suggest biotic nitrogen fixation originated as early as 3.8 to 3.95 Ga and isotopic and genomic evidence supports nitrogen fixation using molybdenum-based nitrogenase occurring as early as 3.2 Ga^7–15^. Whether abiotic or biotic-sourced, continuous flux of N_R_ to early habitats would have been essential for the origin and proliferation of life. These transport mechanisms (e.g., subduction, runoff, atmospheric deposition, hydrothermal interactions, water–air flux) are as important as the source of N_R_ itself.

The isotopic composition of nitrogen in the rock record provides evidence of paleo N_R_ sources and processing at the time of deposition and allows reconstructions of plausible evolutionary pathways for Earth's biogeochemical N cycle. Focusing on the Archean (4.0 to 2.5 Ga), the δ^15^N values in the rock record vary greatly (− 10.2 to + 50.4‰) (Supplementary Fig. 1)^16^ and researchers have proposed various mechanisms, both biotic and abiotic, which would have produced this range. Stüeken et al., Ader et al., and references therein^16,17^, provide a detailed account of potential scenarios which resulted in the Archean data set. In summary, the lower δ^15^N values (down to − 4‰) in the Paleoarchean (4.0 to 3.2 Ga) have been described as representing NH_4_^+^ assimilation by thermophilic microbes, metasomatic alteration, or biological N_2_ fixation via V or Fe nitrogenases (rather than Mo) while higher δ^15^N values (up to 12.2‰) were interpreted as metamorphic overprinting or incomplete biological processing^17,18^. During the Mesoarchean (3.2 to 2.8 Ga), a tight range in δ^15^N values (+ 1.1 ± 1.9‰) across the majority of the data has been suggested as representing Mo N-fixation^11,19^, thus evidencing the nitrogenase enzyme had evolved by this period. The noted excursion from these consistent values is the very high δ^15^N values (up to 50‰) at the approximate transition of the Meso- to Neoarchean era. These high values could not be explained by potential post-depositional alteration and was originally interpreted as reflecting the onset of nitrification and its fractionation effects^20^. A subsequent investigation proposed the high δ^15^N values are a product of large fractionation associated with volatized NH_3_ from alkaline lakes^21^. The consistently positive δ^15^N values across the Neoarchean (2.8 to 2.5 Ga) data set along with ancillary evidence of oxygenation are purported to implicate the onset of competing nitrification and denitrification during this period.

These are plausible explanations for the existing Archean N isotope rock record, which is focused on marine and lacustrine sedimentary rocks. No nitrogen isotopic studies have so far been conducted on Archean terrestrial hot springs, although such sites may have been crucial for the origin and early evolution of life. Here, I present a theoretical framework for nitrogen cycling in such terrestrial hydrothermal settings. I find that NH_3_ outgassing could have produced a wide range of δ^15^N, largely driven by temperature rather than pH. I employ a large data set of the chemical and physical components of modern hydrothermal features to produce evidence that the water–air NH_3_ flux of equivalent Archean features could have been a significant source of bioavailable N to Archean life in neighboring sites that were NH_3_-undersaturated and thus able to absorb NH_3_ from the atmosphere. This water–air flux could be a key component of early Earth’s chemical reactor in which separate environmental settings were linked through physical exchange of reactants and products^2,22^.

## Results and discussion

### Water–air ammonia flux of modern hydrothermal features

**Figure 1:**
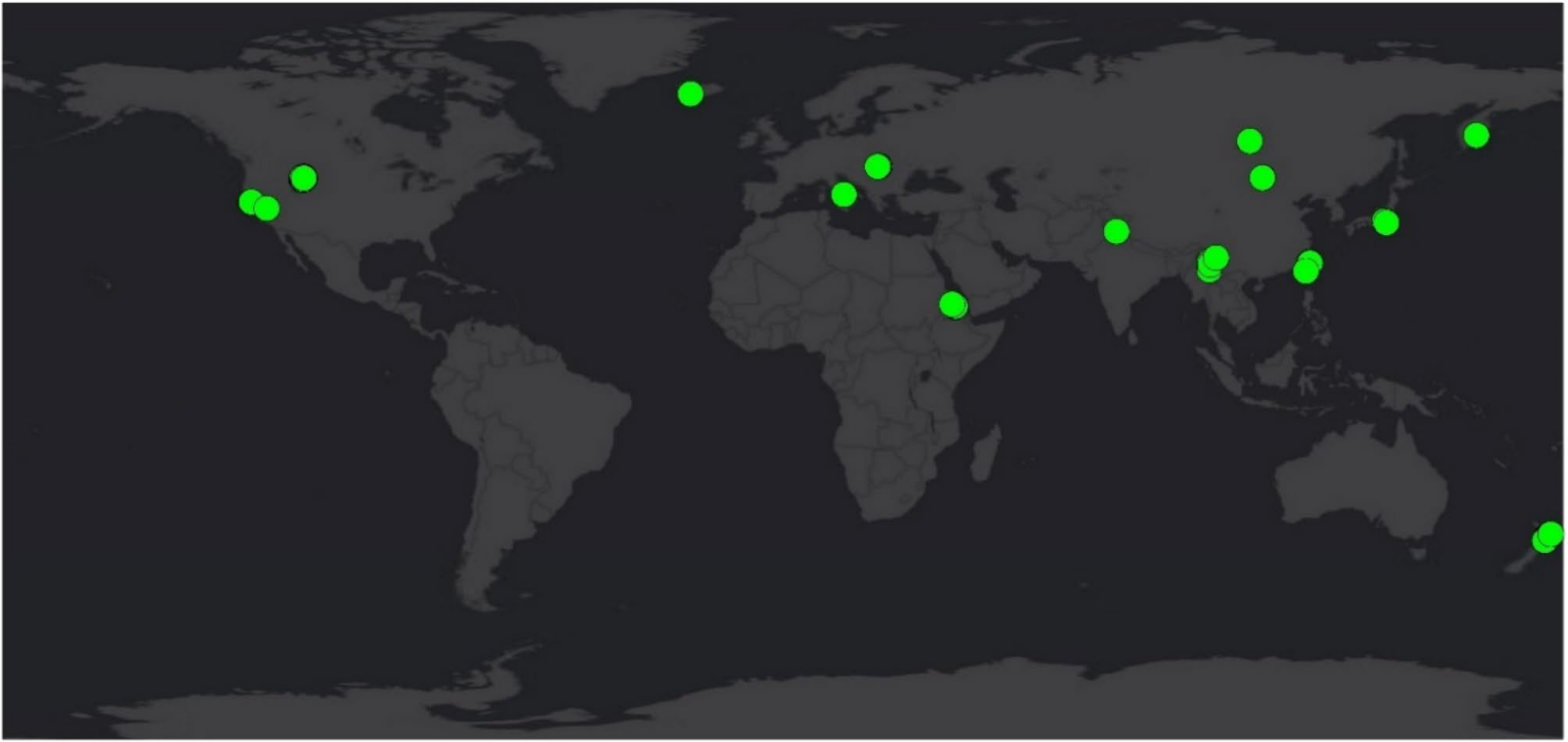
Locations of the 1022 hydrothermal features used as modern analogues to estimate Archean water–air NH_3_ flux. Data and references are included in the Supplemental Information.

**Figure 2:**
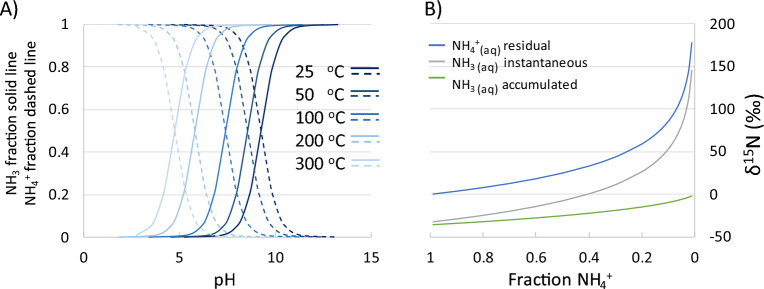
(**A**) Fraction of NH_3_ and NH_4_^+^ in aqueous solution with respect to pH and temperature. (**B**) The δ^15^N values of NH_4_^+^_(aq) residual_, NH_3(aq) instantaneous_, and NH_3(aq) accumulated_ and are modeled with respect to Rayleigh fractionation and the fractionation effect (35.6‰) associated with the average temperature of the observed hydrothermal features (333 Kelvin). Additional less significant fractionation occurs during outgassing (NH_3(aq)_ to NH_3(g)_) and is not included in the model here but the effect is calculated as 6.5‰ at 333 Kelvin (SI)^30^. A starting NH_4_^+^ pool of 0‰ was chosen as the δ^15^N in sedimentary rocks may be representative of the N_2_ fixing organisms present in the early Archean and the δ^15^N of the sedimentary record between 3.2 and 2.5 Ga falls around values of 0‰^31^.

The water–air NH_3_ flux of 1022 hydrothermal features (terrestrial hot springs) across the globe were modeled according to previous approaches^23–27^ using available literature values of NH_4_^+^_(aq)_, salinity, pH, temperature, wind speed and NH_3_ atmospheric concentrations (SI Methods; Supplementary Table 1, Fig. 1). Hydrothermal settings can lead to aqueous systems with wide ranging chemical and physical characteristics which are well represented in the data set. Temperature, pH, salinity and NH_4_^+^ concentration ranges were 298 to 443 K (332 ± 20), 1.2 to 9.7 (4.9 ± 2.3), 0 to 101 ppt (1.4 ± 4.3) and 0 to 46,400 (555 ± 2,764 µM), respectively. Modeled NH_3_ fluxes ranged from − 0.003 to 626 kg N m^−2^ year^−1^ with negative values indicating a NH_3_ flux into the feature and positive values indicating a flux to the atmosphere. The average flux was 2.1 kg N m^−2^ year^−1^, with an outlier removed average flux of 1.2 kg N m^−2^ year^−1^ (outliers were determined using inter-quartile range) and the lower 95% of data average of 0.2 kg N m^−2^. The ability of hot springs to produce these high NH_3_ fluxes have been previously evidenced from the NH_3_ concentration of gases collected directly from two springs in Yellowstone Park, USA (0.1 and 0.7 mol% NH_3(g)_)^28,29^. NH_3_ fluxes are often associated with alkaline waters as a higher pH increases the amount of NH_4_^+^ partitioning to NH_3_. However, with widely varying T the portion of NH_3_ available to flux can be significant even at low pH (Fig. 2A). For instance, at 25 °C and 100 °C the pH at which the NH_4_^+^ to NH_3_ ratio is 50:50 is 9.2 and 7.4, respectively. If this 75 °C increase is applied across the data set, the average flux increases by a factor of 4.2. This has been recounted in a previous sensitivity study which reports T as the primary parameter driving NH_3_ water–air flux^24^. For comparison, if a similar simple sensitivity test is performed on this data set where pH for all sites is increased by 1 unit (i.e., 1 order of magnitude for [H^+^]), the average flux only increases by a factor of 1.3. While previous studies have pointed to a need for alkaline conditions to increase available NH_3_, temperature can greatly increase available NH_3_ and alkaline conditions are not a prerequisite for water–air flux.

To portray the significance of the modeled flux from these hydrothermal features, I determined the portion of Archean Earth surface required to be covered by terrestrial hydrothermal features in order for the mean and outlier-removed mean water–air flux to equal the estimated (1) Phanerozoic land to ocean N flux, (2) modern rock weathering N flux, (3) “modern pre-industrial” atmospheric deposition flux and (4) modern biological fixation flux (Table 1). With respect to modeled outlier removed average flux (1.2 kg N m^−2^ year^−1^), the Archean Earth’s surface would need to be 0.017% hydrothermal features to match the amount of biogenic N fixation on modern Earth. For reference, the combined area of these surficial features would be equivalent to the size of Serbia or the state of Minnesota, USA; a feasible scale relative to the volcanic and hydrothermal activity which would have been widespread during periods of the Archean^32^. While this flux could be seen as a first order loss of bioavailable N from the hot spring environment, the transport and deposition to other environments would significantly complement previously hypothesized abiotic and biotic source mechanisms to provide bioavailable N on early Earth. The transport of this fluxed NH_3_ would be most impactful in nearby terrestrial or aquatic environments due to the direct relationship between concentration and deposition flux (i.e., F = νd × [NH_3_]). Terrestrial hydrothermal features with large NH_3_ water–air flux would create an atmosphere higher in NH_3_ and adjacent undersaturated hot springs or other aquatic settings would readily act as a sink for NH_3_. For instance, the modern ocean can act as a source or sink of NH_3_^24,25,33^ and an Archean ocean adjacent to hot springs may have acted as a sink. Long distance transport of NH_3_ flux may have been hampered due to UV photolysis but this is dependent on the potential presence of a protective organic haze which may have shielded NH_3_ from photolysis^34–36^. This is a scenario which is still debated in the literature^2,7,32^.

**Table 1:** Water–air flux NH_3_ estimates and the corresponding percentage of Archean Earth coverage needed for the flux to equal Phanerozoic N land-to-ocean export flux and modern rock weathering, biological fixation and pre-Industrial Revolution atmospheric deposition N flux.

	NH_3_ water–air flux (kg N m^−2^ year^−1^)	% Earth to equal Phanerozoic land to ocean N flux (2.1 Tg/year)^37,38^	% Earth to equal modern rock weathering N flux (15.1 Tg/year)^39^	% Earth to equal modern bio fix N flux (100.1 Tg/year)^39^	% Earth coverage to equal pre-industrial atm dep N flux (11.2 Tg/year)^39^
Range	− 0.003 to 626	N sink to 5.4 × 10^–7^%	N sink to 4.7 × 10^–6^%	N sink to 3.1 × 10^–5^%	N sink to 3.5 × 10^–6^%
Mean	2.1	1.9 × 10^–4^%	0.0014%	0.0092%	0.0010%
Mean, outliers removed	1.2	3.6 × 10^–4^%	0.0026%	0.017%	0.0019%
Lower 95%	0.2	21.6 × 10^–4^%	0.0156%	0.102%	0.0114%

Archean hydrothermal waters would have been NH_4_^+^ poor and unable to produce a large NH_3_ flux unless they had circulated through sedimentary formations featuring older organic matter^40,41^. For the water–air NH_3_ flux to be as pronounced as those from modern analogs, previous biotic and/or abiotic processes would have needed to supply substantial NH_4_^+^ to Paleoarchean sediments. As abiotic sources were estimated to be too low to produce enough N_R_ for early life and subsequent organic deposition, the biotic source previously substantiated by high NH_4_^+^ phyllosilicates and isotopic and genomic evidence of nitrogen fixers in the Archean would have been required by the Paleoarchean. This would mean biotic-sourced N that was previously assimilated and deposited as organic matter in lithified sediments would be supplied to the features through hydrothermal remobilization in quantities large enough to produce significant water–air NH_3_ fluxes. This mechanism creates up to millimolar NH_4_^+^ concentrations found in modern hot springs and previous studies date this hydrothermal recycling process of organic bound N back to at least 3.24 Ga^42^. It must be noted that these Archean studies refer to hydrothermal cycling in the deep ocean so is not directly applicable to terrestrial hot springs but rather provides evidence for hydrothermal recycling of organic bound during this period.

### Potential isotopic signature for hydrothermal water–air ammonia flux

The early Earth rock record has a wide range of δ^15^N (− 10.2 to 50.4‰) compared to the tighter range in modern sediments that is attributed to biological mediation (Supplementary Fig. 1). The wide range is a product of competing abiotic and biotic processes with the higher end values ascribed to incomplete biological N processing and/or NH_3_ volatilization from alkaline paleolakes^20,21^. With respect to rock records potentially produced in terrestrial hydrothermal settings, here, I present evidence that a wide range of δ^15^N can be a result of fractionation effects associated with terrestrial hydrothermal features and processes. For instance, a previous isotopic investigation of modern hot springs reports a similar range of δ^15^N-NH_4_^+^ values in water samples and sediment samples of − 6 to 30‰ and − 10 to 23‰, respectively^28^. The higher portion of the δ^15^N range in these modern springs was produced by preferential partitioning and volatilization of ^14^NH_3_. Deng et al., state these systems are not static and can be regarded and modeled as open system^30^. The isotope effects are modeled (Fig. 2B) for a hypothetical Archean hot spring using a starting δ^15^N-NH_4_^+^_(aq)_ value of 0‰ and a temperature-dependent isotope effect of 35.6‰ which corresponds to the average temperature of the modern hydrothermal features (333 K) (SI Methods)^43^. A starting NH_4_^+^ pool of 0‰ was chosen as the δ^15^N in sedimentary rocks as it would be representative of the N_2_ fixing organisms present in the early Archean in which a majority of δ^15^N values fall around 0‰^31^. Interaction between hot fluids and organic-bound N imparts minimal isotopic fractionation as N is remobilized^44^. Additional, less significant fractionation, which would further enrich the aqueous NH_3_, occurs during outgassing (NH_3(aq)_ to NH_3(g)_) and is not included in the model here but for reference the effect is calculated as 6.5‰ at 333 K^30^. Model results display that the δ^15^N of the residual NH_4_^+^ which would subsequently be imprinted in the rock record encompasses the higher end positive δ^15^N represented in the Archean rock record, although it should be stressed that there is no known evidence of hydrothermal activity in the Neoarchean Tumbiana Formation where the highest δ^15^N values have been recorded. The scenario invoked herein may, however, apply in other, hydrothermally active settings on Archean land surfaces. The higher δ^15^N values would be concentrated in the immediate depositional environment while the lighter product of this volatilization would be deposited elsewhere, mixing with large pools of N and potentially lowering its δ^15^N. This scenario was suggested by Holloway et al.^28^ in which they explain the modern springs with lower δ^15^N values contain NH_4_^+^ derived from recondensation of ^15^N-depleted NH_3_(g) that was transported from sites of phase separation and partial NH_4_^+^ loss. These isotope effects followed by deposition and mixing with other NH_4_^+^ pools would lead to a wide range of δ^15^N depending on environmental settings and existing N pools.

Hadean and Archean volcano-hydrothermal systems were estimated to be 4 to 5 times more active than present day due to radiogenic heating^32^ lending the Archean Earth to having more prevalent hydrothermal features. While many of these features may have been submarine due the early Archean Earth resembling a “water world”^45,46^, modeled NH_3_ flux results reported here suggest only 0.0092% of the Earth is needed to host terrestrial hydrothermal features to rival the significance of present-day biogenic fixation. While investigations based on siliciclastic sediments propose some continental exposure as early as 3.8 billion years ago^47^, low but more pronounced exposure in the Meso- and Neoarchean is indicated by paleosol and Sr isotope studies^45,48,49^. Evidence of continental crust growth throughout the Archean, particularly associated with island arcs^50,51^, suggests a potential setting for the emergence of associated terrestrial hydrothermal features. These features may have been linked with hydrothermal systems circulating organic bound N in marine sediments, subsequently providing NH_3_ for water–air flux. A portion of the higher and wide ranging δ^15^N values in the Archean rock record are observed in the Pilbara Supergroup where hydrothermal features were a common environment including hot springs and hydrothermal vents^52,53^. While this specific nitrogen isotope fractionation pathway described in this study can so far not be linked to the existing δ^15^N record from the Archean as evidence of hydrothermal processes is lacking for sites with unusual δ^15^N enrichments, other formations, for instance the Dresser formation, Pilbara Craton, show evidence of surficial hydrothermal features including geyserite, sinter terracettes and mineralized remnants of hot spring pools/vents^54^ and may prove to have recorded these processes. However, it should be noted origins of features in this formation are still under debate and could be remnants of deep-sea hydrothermal systems^55,56^. While the ^15^N rock record data from the Archean is limited, this eon was affected by widespread volcanism and the associated hydrothermal systems interacting with N enriched lithified sediments could have produced a setting prime for water–air flux of bioavailable N to the Archean atmosphere.

### Implications for early Earth N_R_ processing

Hydrothermal features (e.g. hot springs) provide a concentrated source of diverse chemical species and an expansive range of physical environments to support a wide array of microbial communities^57^. These characteristics have led many researchers to investigate hot springs as an ideal setting for the origin of life. Experiments have shown key feedstock molecules for prebiotic chemistry can be produced in abundance in shallow and surficial hydrothermal systems^58^. This coupled with evidence that hydrothermal fluids actively leached Mo, a strict trace metal requirement for Mo-nitrogenase, from volcanic rocks in the Archean support hydrothermal environments as a potential origin of biological N_2_ fixation^15,59–62^. Regardless of whether life originated in these hydrothermal features, the NH_3_ flux from hot NH_3_-supersaturated springs to nearby cooler, NH_3_-undersaturated springs may have supplied the essential bioavailable N in these cooler settings to spur and sustain life. Characterizing the NH_4_^+^ source and transport mechanisms is fundamental to reconstructing the N cycle during the Archean, a period before widespread established oxygenic photosynthesis meant the cycle was driven by the interplay between diazotrophy, ammonium regeneration and assimilation^7^. Well preserved Archean sediments are not common, and of the few Archean rock record studies, the majority have been concentrated on low energy, deeper water settings. Future sampling focused on paleo-hydrothermal settings, including those younger than the Archean will be valuable in assessing the validity of these hydrothermal feature effects on the range of δ^15^N in the rock record. Including corroborating data (e.g., trace metals, Fe speciation redox indicators, C/N ratio, δ^13^Corg and δ^13^Ccarb and biomarker δ^15^N) in these studies will be essential to predict paleo-depositional environments and potential mechanisms creating the δ^15^N rock signatures^63^. These additional diagnostic tools could provide localized evidence of settings for significant water–air NH_3_ flux on early Earth.

I do recognize, as with most studies attempting to predict the production, transport and processing of N on early Earth, the Archean water–air flux estimates are based on the assumption that modern analogs are a reasonable proxy. The multiple lines of evidence presented in this work attempt to support this modern analog and suggest that the water–air NH_3_ flux was a prominent mechanism providing bioavailable N to neighboring NH_3_-undersaturated environments as part of the early Earth’s chemical reactor and may even be underestimated as the presence of O_2_ in the modern environment would reduce the amount of NH_3_ available for outgassing. Potential applications outside the scope of this current work include investigating whether this flux is substantial enough to produce a greenhouse gas effect to counteract the Faint Young Sun and whether remote sensing could be used to detect this potential biogenic signal on exoplanets^64^. The proposed δ^15^N rock signature originating from terrestrial hydrothermal features holds significant potential for the identification of analogous past and current features on other planets and their satellites. Geologically active satellites in our solar system, such as Europa, Ganymede, Enceladus and Titan, provide intriguing possibilities of hydrothermal systems and the presence of past water on Mars, combined with compelling geomorphological and mineral evidence, indicates the occurrence of previous hydrothermal activity^65,66^. For instance, the morphology of opaline silica deposits located near the Home Plate feature in the Columbia Hills of Gusev crater on Mars strongly suggests they are the product of sinter deposits resulting from hot spring activity^67^. However, the origin of these deposits remains a subject of ongoing debate. Researchers should consider this hydrothermal flux mechanism along with other hypothesized abiotic and biotic processes when applying the δ^15^N record to unravel the development of Earth’s biogeochemical N cycle and explore potential biosignatures on other planets.

## Methods

A literature search was conducted via google scholar to obtain hydrothermal feature data which included the NH_4_^+^ concentration, salinity, temperature and pH needed to model the NH_3_ water–air flux. These prerequisite data were met for 1022 features (data and references in Supplementary Table 1) and flux calculations were based on previous models^15–19^. The direction and magnitude of the water–air flux of NH_3_ can be determined from the atmospheric concentration of NH_3_, a calculated atmospheric equilibrium concentration of NH_3_ and an exchange velocity. The difference between the atmospheric and equilibrium NH_3_ concentrations provides the direction of flux, with a positive value denoting water-atmosphere NH_3_ emission from the feature and a negative value denoting NH_3_ deposition to the feature. By multiplying that difference by an air-side exchange velocity, a rate of water-atmosphere NH_3_ flux can be determined^19^ (Eq. 1):

1 $${\text{F}}_{{{\text{NH3}}}} = {\text{ kg }} \times \, \left[ {{\text{NH}}_{{3}} \left( {{\text{eq}}} \right) \, {-}{\text{ NH}}_{{3}} \left( {\text{g}} \right)} \right].$$

F_NH3_ is the water-atmosphere NH_3_ flux (ng m^−2^ s^−1^), kg is the air-side exchange velocity (m/s), NH_3_(eq) is the calculated atmospheric equilibrium NH_3_ concentration (µg/m^3^), and NH_3_(g) is the atmospheric NH_3_ concentration (µg/m^3^). Full details about how each term was determined are located in SI.

### Supplementary Information

Supplementary Information 1.

Supplementary Information 2.

## Data Availability

All data is available in the main text or within the Supplemental Documents.
