# Supplementary material for: Modern analogs for ammonia flux from terrestrial hydrothermal features to the Archean atmosphere
Source: Sci Rep. 2024 Jan 17;14:1544. doi: 10.1038/s41598-024-51537-2 (PMC10794450; doi:10.1038/s41598-024-51537-2)
Supplement: Supplementary file 1 — Supplementary Information 1. [file 41598_2024_51537_MOESM1_ESM.docx]

**This supplementary file contains supplementary methods and a table legend for a supplementary table.**

**1. Supplementary Methods**

**1.1 Data compilation for modeling water-atmosphere NH_3_ flux**

**1.2 Determining water-atmosphere NH_3_ flux**

**1.3 Calculating the NH_4_^+^_(aq)_ → NH_3(aq)_ and NH_3(aq)_ → NH_3(g)_ temperature-dependent isotope effect Table SI 1. Literature data compiled to calculate air-water flux. Excel sheet included in supplemental materials.**

**1. Supplementary Methods**

**1.1 Data compilation for modeling water-atmosphere NH_3_ flux**

A literature search was conducted via google scholar to obtain hydrothermal feature data which included the NH_4_^+^ concentration, salinity, temperature and pH needed to model the NH_3_ air-water flux. These prerequisite data were met for 1022 features (Table SI 1). For other parameters needed, ammonia monitoring networks are still developing worldwide so atmospheric ammonia concentrations were not readily available at each site. The majority of these sites are located in rural areas denoted as National Parks. The US National Atmospheric Deposition Program’s Ammonia Monitoring Network has samplers in Yellowstone National Park, Wyoming, US (AMoN sites WY92 and WY94) ^1^. The average concentration at these sites (0.35 µg/m^3^) was used as a plausible representative for the atmospheric concentration at the hydrothermal feature sites. Wind speed is the average annual wind speed calculated from publicly available meteorological data in each site’s region. When salinity was not directly available but Cl^-^ mg/L was multiplied by a conversion factor of 0.0018066 to estimate salinity ^2^.

**1.2 Determining water-atmosphere NH_3_ flux**

Since we cannot conduct direct flux measurements at over 1022 hydrothermal features across the globe, we used literature data and previous modeling approaches to calculate the air-water flux. The direction and magnitude of the air-water flux of NH_3_ can be determined from the atmospheric concentration of NH_3_, a calculated atmospheric equilibrium concentration of NH_3_ and an exchange velocity^3, 4, 5, 6, 7^ (e.g. Asman et al., 1994; Quinn et al., 1988, 1996; Johnson et al. 2008; Wentworth et al., 2016). The difference between the atmospheric and equilibrium NH_3_ concentrations reveals the direction of flux, with a positive value denoting water-atmosphere NH_3_ emission and a negative value denoting NH_3_ deposition. By multiplying that difference by an air-side exchange velocity, a rate of water-atmosphere NH_3_ flux can be determined ^7^(Wentworth et al., 2016) (Equ SI1):

F_NH3_ = k_g_ * [NH_3(eq)_ – NH_3(g)_] Equ S1.

F_NH3_ is the water-atmosphere NH_3_ flux (ng m^-2^ s^-1^), k_g_ is the air-side exchange velocity (m/s), NH_3(eq)_ is the calculated atmospheric equilibrium NH_3_ concentration (µg/m^3^), and NH_3(g)_ is the atmospheric NH_3_ concentration (µg/m^3^). As presented by Wentworth et al. (2016) from McKee (2001), the exchange velocity k_g_ is determined as:

k_g_ = *w* / 770 + [45 * (17.0305^1/3^)], Equ. S2

where *w* is the measured wind speed (m/s). The atmospheric equilibrium concentration (NH_3(eq)_) is found by:

NH_3(eq)_ = NH_3(aq)_ * *K*_H_ Equ. S3

where NH_3(aq)_ is the concentration of aqueous NH_3_ and *K*_H_ is the dimensionless Henry’s Law constant (Wentworth et al., 2016). The following calculation is performed to estimate the proportion of NH_3_ of total ammonia (NH_X_) in the water:

NH_3(aq)_ = NH_X_ * P_NH3_ Equ. S4

where P_NH3_ is the NH_3_ proportion factor derived from:

P_NH3_ = *K*_a_ / (*K*_a_ + H^+^) Equ. S5

Here, *K*_a_ is the acid dissociation factor as determined from the acid dissociation coefficient (p*K*_a_) of ammonium in marine water, and H^+^ is the concentration of hydrogen ions as determined from the measured pH of the water ^7, 8^ (Bell at al., 2007; Wentworth et al., 2016):

H^+^ = 10^-pH^ Equ S6
*K*_a_ = 10^-p^*^K^*^a^ Equ S7

To calculate p*K*_a_, the measured water temperature (*t*) in °C and salinity (S) in ppt are needed (Bell at al., 2007):

p*K*_a_ = 10.0423 – (0.0315536 * *t*) + (0.003071 * S) Equ S8

Finally, the measured water temperature (T) in kelvin is utilized to determine the Henry’s Law constant (Wentworth et al., 2016):

*K*_H_ = 1 / [17.93 * (T / 273.15) * *e*^(4092/T)-9.70^] Equ S9

By the mathematical model outlined above, measurements of wind speed, water temperature, pH and salinity, and calculations of the atmospheric and water concentrations of NH_3_ are applied to produce an estimate of the water-atmosphere flux of NH_3_.

**1.3 Calculating the NH_4_^+^_(aq)_ → NH_3(aq)_ and NH_3(aq)_ → NH_3(g)_ temperature-dependent isotope effect**

Li et al., used empirical data from laboratory experiments to model the relationship between equilibrium isotopic fractionation between NH_4_^+^_(aq)_ and NH_3(aq)_ and temperature as 10^3^ ^.^ lnα_NH4+_ - _NH3_ _(aq)_ = 25.94 x 10^3^/T – 42.25^9^. Using this relationship, the temperature-dependent isotope effect at the average temperature of the modern hydrothermal features (333 Kelvin) was calculated to be 35.6‰.

Deng et al., used empirical data from laboratory experiments to model the fractionation between NH_3(aq)_ and NH_3(g)_ during outgassing with respect to temperature as 10^3^ ^.^ lnα_NH3+(aq)_ - _NH3_ _(g)_ = 14.6 – 6.8 x 10^3^/T ^10^. Using this relationship, the temperature-dependent isotope effect at the average temperature of the modern hydrothermal features (333 Kelvin) was calculated to be 6.5‰.

**
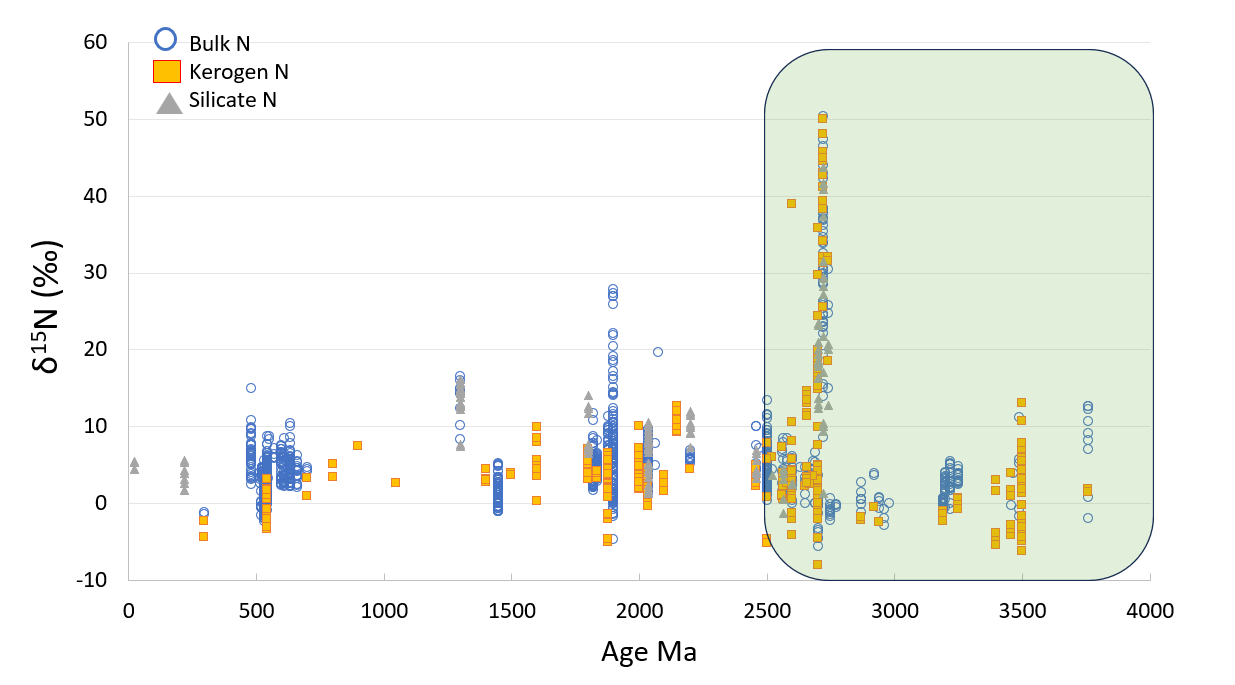
**

**Figure SI 1.** δ^15^N values of bulk, kerogen and silicate nitrogen in the rock record primarily obtained from Ader et al., 2016 supplemental information and references therein ^11, 12, 13^. The shaded portion of the graph represents the period of focus for this study.

**Table SI 1.** Literature data of chemical and physical hydrothermal features compiled to calculate air-water flux. Excel sheet included in supplemental materials. References below. ^14, 15, 16, 17, 18, 19, 20, 21, 22, 23, 24, 25, 26, 27, 28, 29, 30, 31^


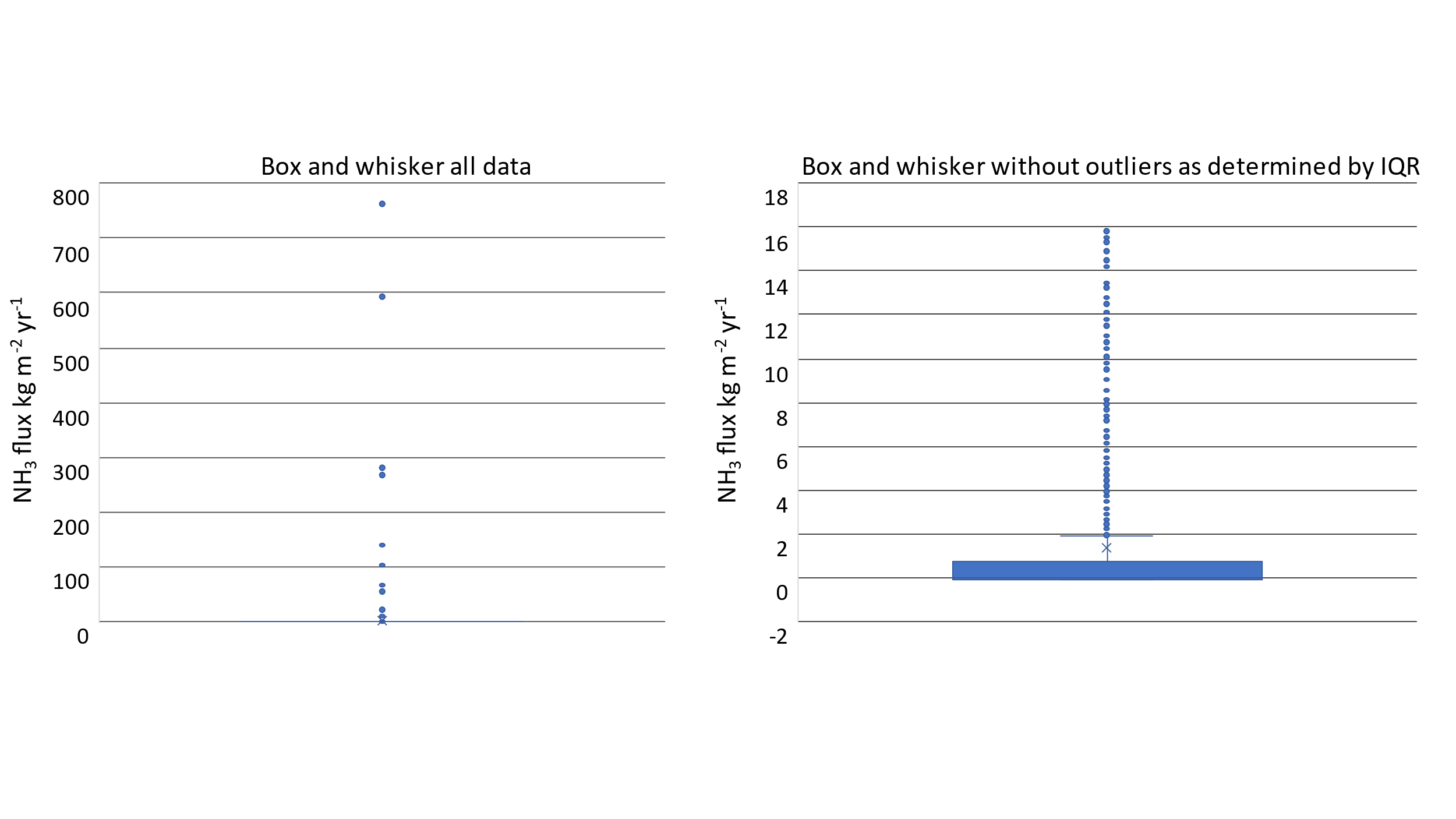


**Figure SI 2.** Left) Box and whisker plot of modeled NH_3_ flux for each hot spring. Right) Box and whisker plot of modeled NH_3_ flux without outliers as determined by IQR.


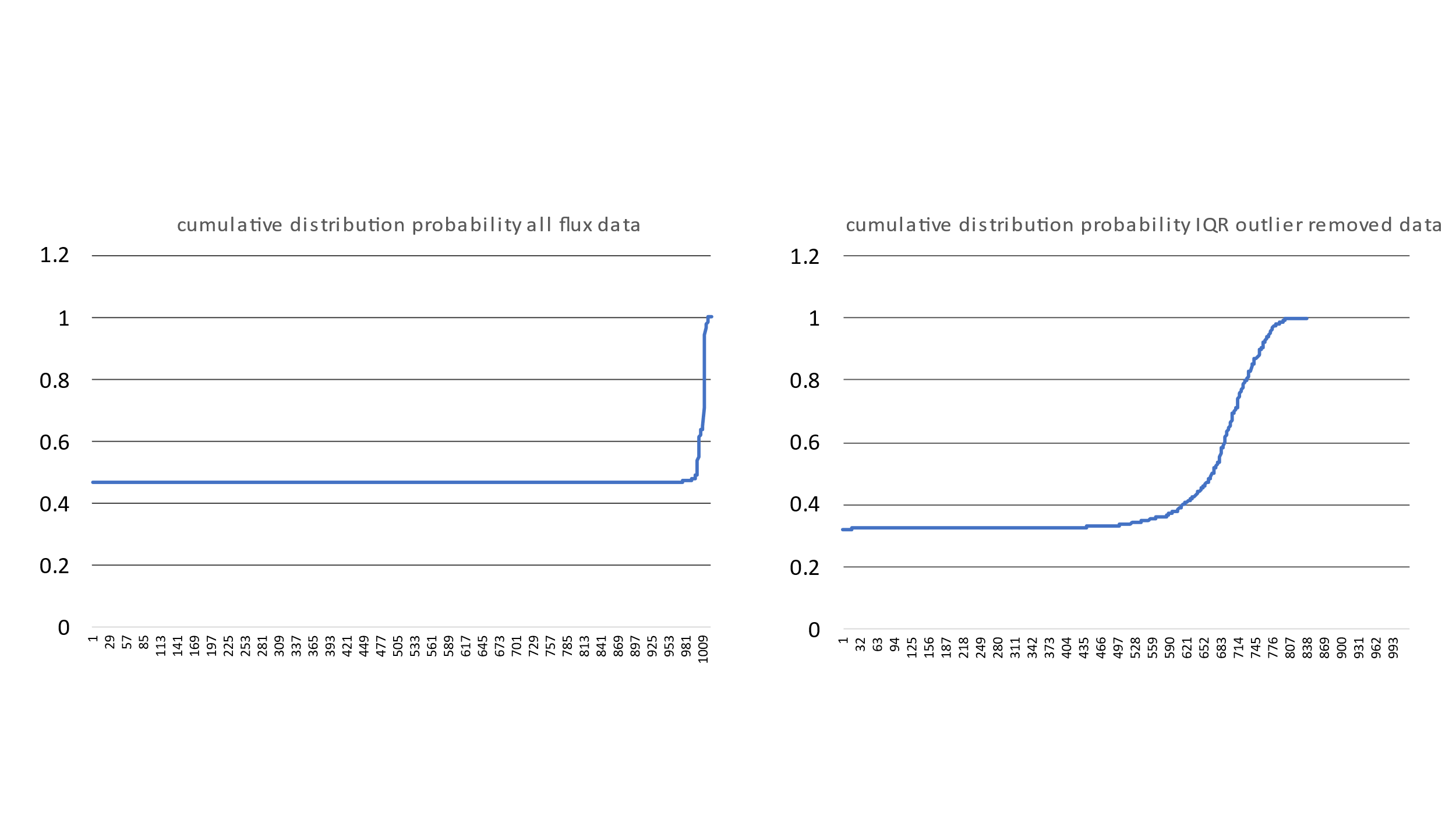


**Figure SI 3.** Left) Cumulative distribution probability all flux data. Right) Cumulative distribution probability of IQR outlier removed data.

**References**

1. NADP. National Atmosphric Deposition Program Ammonia Monitoring Network. [*https://nadpslhwiscedu/networks/ammonia-monitoring-network/*](https://nadpslhwiscedu/networks/ammonia-monitoring-network/) 2022.

2. Brankovits D, Pohlman JW, Ganju NK, Iliffe TM, Lowell N, Roth E*, et al.* Hydrologic Controls of Methane Dynamics in Karst Subterranean Estuaries. *Global Biogeochemical Cycles* 2018, **32**(12)**:** 1759-1775.

3. Asman W, Harrison R, Ottley C. Estimation of the air-sea flux of ammonia over the southern bight of the north sea. *Atmospheric Environment* 1994, **28**(22)**:** 3647-3654.

4. Quinn P, Barrett K, Dentener F, Lipschultz F, Six K. Estimation of the Air/Sea Exchange of Ammonia for the North Atlantic Basin. *Biogeochemistry* 1996, **35**(1)**:** 275-304.

5. Quinn P, Charlson R, Bates T. Simultaneous observations of ammonia in the atmosphere and ocean. *Nature* 1988, **335**.

6. Johnson MT, Liss PS, Bell TG, Lesworth TJ, Baker AR, Hind AJ*, et al.* Field observations of the ocean-atmosphere exchange of ammonia: Fundamental importance of temperature as revealed by a comparison of high and low latitudes. *Global Biogeochemical Cycles* 2008, **22**(1)**:** n/a-n/a.

7. Wentworth GR, Murphy JG, Croft B, Martin RV, Pierce JR, Côté J-S*, et al.* Ammonia in the summertime Arctic marine boundary layer: sources, sinks, and implications. *Atmospheric Chemistry and Physics* 2016, **16**(4)**:** 1937-1953.

8. Bell TG, Johnson MT, Jickells TD, Liss PS. Ammonia/ammonium dissociation coefficient in seawater: A significant numerical correction. *Environmental Chemistry* 2007, **4**(3).

9. Li L, Lollar BS, Li H, Wortmann UG, Lacrampe-Couloume G. Ammonium stability and nitrogen isotope fractionations for –NH3(aq)–NH3(gas) systems at 20–70°C and pH of 2–13: Applications to habitability and nitrogen cycling in low-temperature hydrothermal systems. *Geochimica et Cosmochimica Acta* 2012, **84:** 280-296.

10. Deng Y, Li Y, Li L. Experimental investigation of nitrogen isotopic effects associated with ammonia degassing at 0–70 °C. *Geochimica et Cosmochimica Acta* 2018, **226:** 182-191.

11. Ader M, Thomazo C, Sansjofre P, Busigny V, Papineau D, Laffont R*, et al.* Interpretation of the nitrogen isotopic composition of Precambrian sedimentary rocks: Assumptions and perspectives. *Chemical Geology* 2016, **429:** 93-110.

12. Yang J, Junium CK, Grassineau NV, Nisbet EG, Izon G, Mettam C*, et al.* Ammonium availability in the Late Archaean nitrogen cycle. *Nature Geoscience* 2019, **12**(7)**:** 553-557.

13. Homann M, Sansjofre P, Van Zuilen M, Heubeck C, Gong J, Killingsworth B*, et al.* Microbial life and biogeochemical cycling on land 3,220 million years ago. *Nature Geoscience* 2018, **11**(9)**:** 665-671.

14. Button M, Cary C. One Thousand Springs. The Microbial Geothermal Hot Springs in New Zealand. *GNS Science and the University of Waikato* 2022, [**https://1000springs.org.nz/**](https://1000springs.org.nz/)

15. Chiriac CM, Szekeres E, Rudi K, Baricz A, Hegedus A, Dragos N*, et al.* Differences in Temperature and Water Chemistry Shape Distinct Diversity Patterns in Thermophilic Microbial Communities. *Appl Environ Microbiol* 2017, **83**(21).

16. Holloway JM, Nordstrom DK, Böhlke JK, McCleskey RB, Ball JW. Ammonium in thermal waters of Yellowstone National Park: Processes affecting speciation and isotope fractionation. *Geochimica et Cosmochimica Acta* 2011, **75**(16)**:** 4611-4636.

17. Chen S, Peng X, Xu H, Ta K. Nitrification of archaeal ammonia oxidizers in a high- temperature hot spring. *Biogeosciences* 2016, **13**(7)**:** 2051-2060.

18. Chen W-F, Sung M. The Redox Potential of Hot Springs in Taiwan. *Terrestrial, Atmospheric and Oceanic Sciences* 2009, **20**(3).

19. Ghilamicael AM, Budambula NLM, Anami SE, Mehari T, Boga HI. Evaluation of prokaryotic diversity of five hot springs in Eritrea. *BMC Microbiol* 2017, **17**(1)**:** 203.

20. Hou W, Wang S, Dong H, Jiang H, Briggs BR, Peacock JP*, et al.* A comprehensive census of microbial diversity in hot springs of Tengchong, Yunnan Province China using 16S rRNA gene pyrosequencing. *PLoS One* 2013, **8**(1)**:** e53350.

21. Ioka S, Muraoka H, Matsuyama K, Tomita K. In situ redox potential measurements as a monitoring technique for hot spring water quality. *Sustainable Water Resources Management* 2016, **2**(4)**:** 353-358.

22. Jiang H, Huang Q, Dong H, Wang P, Wang F, Li W*, et al.* RNA-based investigation of ammonia-oxidizing archaea in hot springs of Yunnan Province, China. *Appl Environ Microbiol* 2010, **76**(13)**:** 4538-4541.

23. Lebedeva EV, Alawi M, Fiencke C, Namsaraev B, Bock E, Spieck E. Moderately thermophilic nitrifying bacteria from a hot spring of the Baikal rift zone. *FEMS Microbiol Ecol* 2005, **54**(2)**:** 297-306.

24. Reigstad LJ, Richter A, Daims H, Urich T, Schwark L, Schleper C. Nitrification in terrestrial hot springs of Iceland and Kamchatka. *FEMS Microbiol Ecol* 2008, **64**(2)**:** 167-174.

25. Roberson C, Whitehead H. Ammoniated Thermal Waters of Lake and Colusa Counties California. 1961, **GEOLOGICAL SURVEY WATER-SUPPLY PAPER 1535-A**(US Government Printing Office).

26. Shock EL, Holland M, Amend J. Geochemical Sources of Energy for Microbial Metabolism in Hydrothermal Ecosystems: Obsidian Pool, Yellowstone National Park. *GEOTHERMAL BIOLOGY AND GEOCHEMISTRY IN YELLOWSTONE NATIONAL PARK* 2005.

27. Singh Y, Gulati A, Singh DP, Khattar JIS. Cyanobacterial community structure in hot water springs of Indian North-Western Himalayas: A morphological, molecular and ecological approach. *Algal Research* 2018, **29:** 179-192.

28. Valentino G, Cortecci G, Franco E, Stanzione D. Chemical and isotopic compositions of minerals and waters from the Campi Flegrei volcanic system, Naples, Italy. *Journal of Volcanology and Geothermal Research* 1999, **91:** 329–334.

29. Vick TJ, Dodsworth JA, Costa KC, Shock EL, Hedlund BP. Microbiology and geochemistry of Little Hot Creek, a hot spring environment in the Long Valley Caldera. *Geobiology* 2010, **8**(2)**:** 140-154.

30. Ward LM, Idei A, Nakagawa M, Ueno Y, Fischer WW, McGlynn SE. Geochemical and Metagenomic Characterization of Jinata Onsen, a Proterozoic-Analog Hot Spring, Reveals Novel Microbial Diversity including Iron-Tolerant Phototrophs and Thermophilic Lithotrophs. *Microbes Environ* 2019, **34**(3)**:** 278-292.

31. Zhao W, Song Z, Jiang H, Li W, Mou X, Romanek CS*, et al.* Ammonia-oxidizing Archaea in Kamchatka Hot Springs. *Geomicrobiology Journal* 2011, **28**(2)**:** 149-159.
